# Supplementary material for: Understanding the impact of SNPs associated with autism spectrum disorder on biological pathways in the human fetal and adult cortex
Source: Sci Rep. 2021 Aug 5;11:15867. doi: 10.1038/s41598-021-95447-z (PMC8342620; doi:10.1038/s41598-021-95447-z)
Supplement: Supplementary file 1 — Supplementary Figures. [file 41598_2021_95447_MOESM1_ESM.pdf]

Supplementary figures for:

Understanding the impacts of SNPs associated with autism spectrum disorder on biological pathways in the human fetal and adult cortex.

Golovina E.[1], Fadason T.[1,2], Lints T.J.[3], Walker C.[4], Vickers M.H.[1,2], O'Sullivan J.M.[1,2,5,6,7*]

[1] Liggins Institute, University of Auckland, New Zealand

[2] Maurice Wilkins Centre, University of Auckland, New Zealand

[3] School of Medical Science, University of Auckland, New Zealand

[4] School of Population Health, University of Auckland, New Zealand

[5] Brain Research New Zealand, University of Auckland, New Zealand

[6] MRC Lifecourse Epidemiology Unit, University of Southampton, United Kingdom

[7] Garvan Institute of Medical Research, Sydney, Australia

* corresponding author: justin.osullivan@auckland.ac.nz

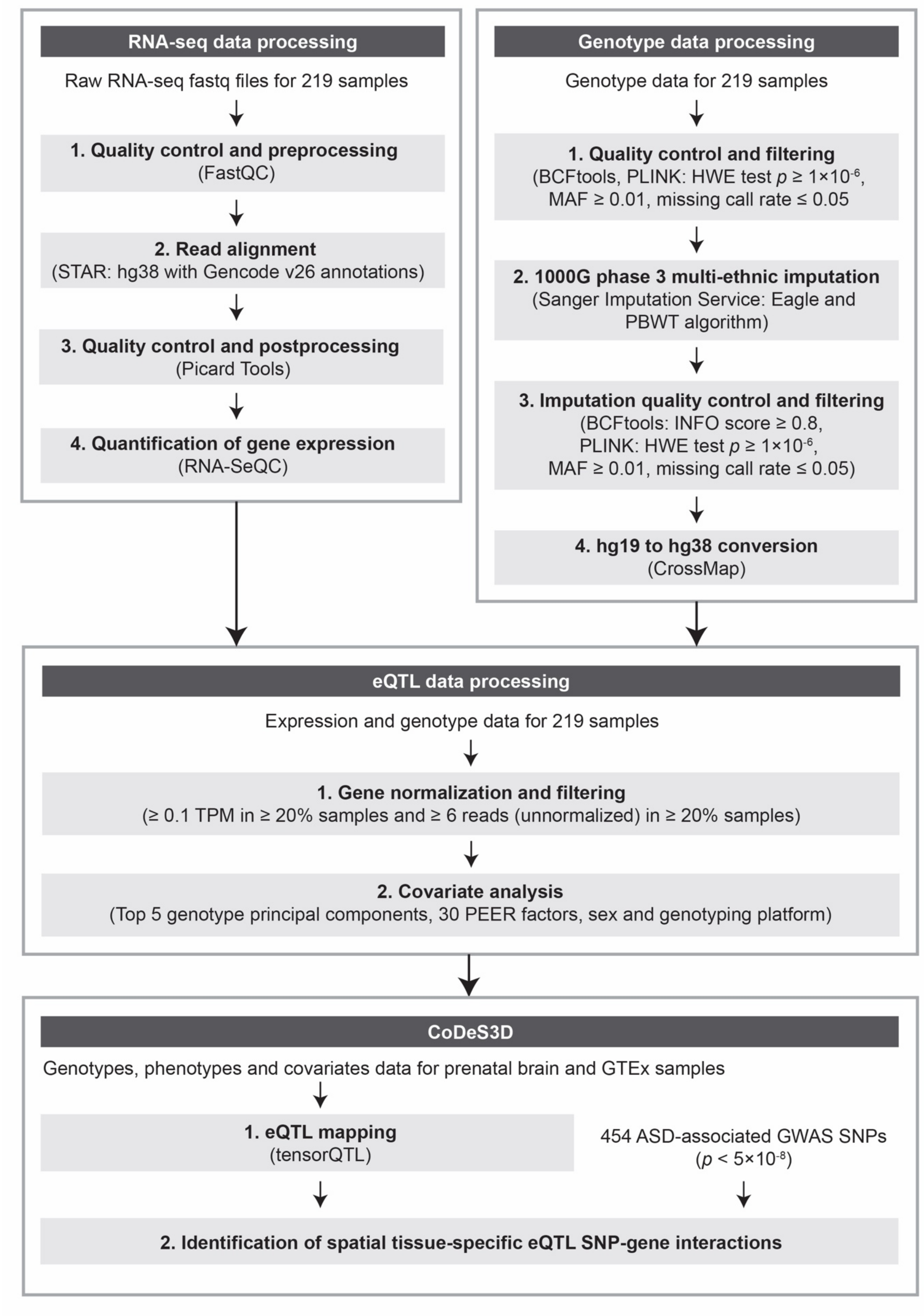

Supplementary Figure 1: Overview of the pipeline that was used for fetal brain RNA sequencing and genotype data processing. Output data from this pipeline were used as inputs

for eQTL analysis and identification of spatial eQTL-gene interactions in the fetal brain, using the CoDeS3D algorithm (Figure 1).

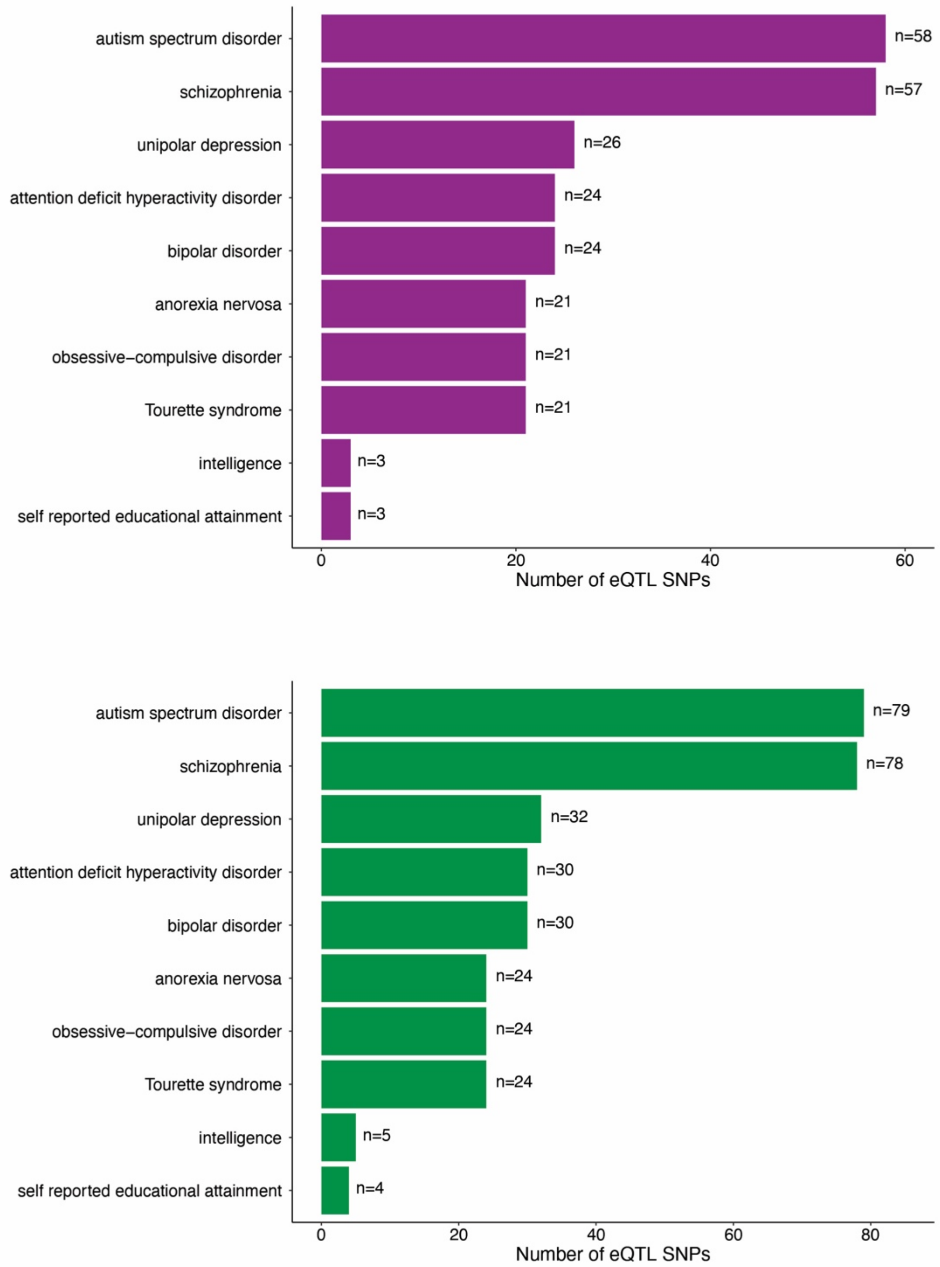

Supplementary Figure 2: Rank order representation of the top 10 GWAS traits ($p < 5\times10^{-8}$, assessed on 26/08/2020) for which the ASD-associated eQTLs are annotated as being risk loci. 57 out of 58 ASD-associated eQTL SNPs in adult cortex and 78 out of 79 eQTL SNPs in fetal cortex were associated with schizophrenia. These overlaps are statistically significant (bootstrapping, $p < 0.01$, n=10,000).

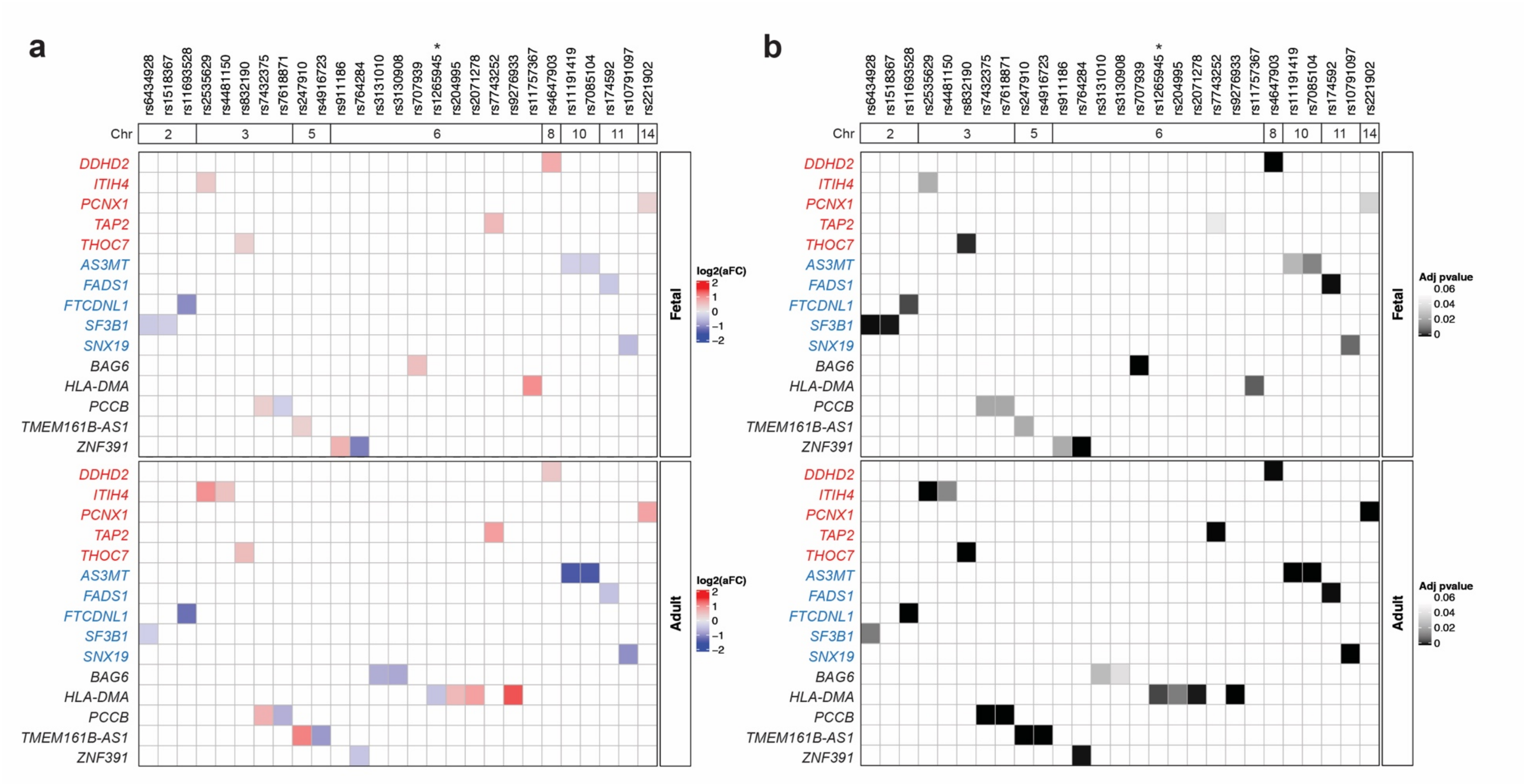

Supplementary Figure 3: The ASD-associated eQTLs typically affected gene transcript levels collinearly up- and downregulate the expression of 15 eGenes common in both adult and fetal cortical tissues. **a.** ASD-associated eQTLs that present in both tissues typically affect gene transcript levels colinearly. However, specific eQTLs that act only in fetal or adult cortical tissue. **b.** Adjusted *p* (adj $p < 0.05$) for the ASD-associated eQTLs in a. *, gene whose transcript levels were associated with a trans-acting ASD-associated eQTL.

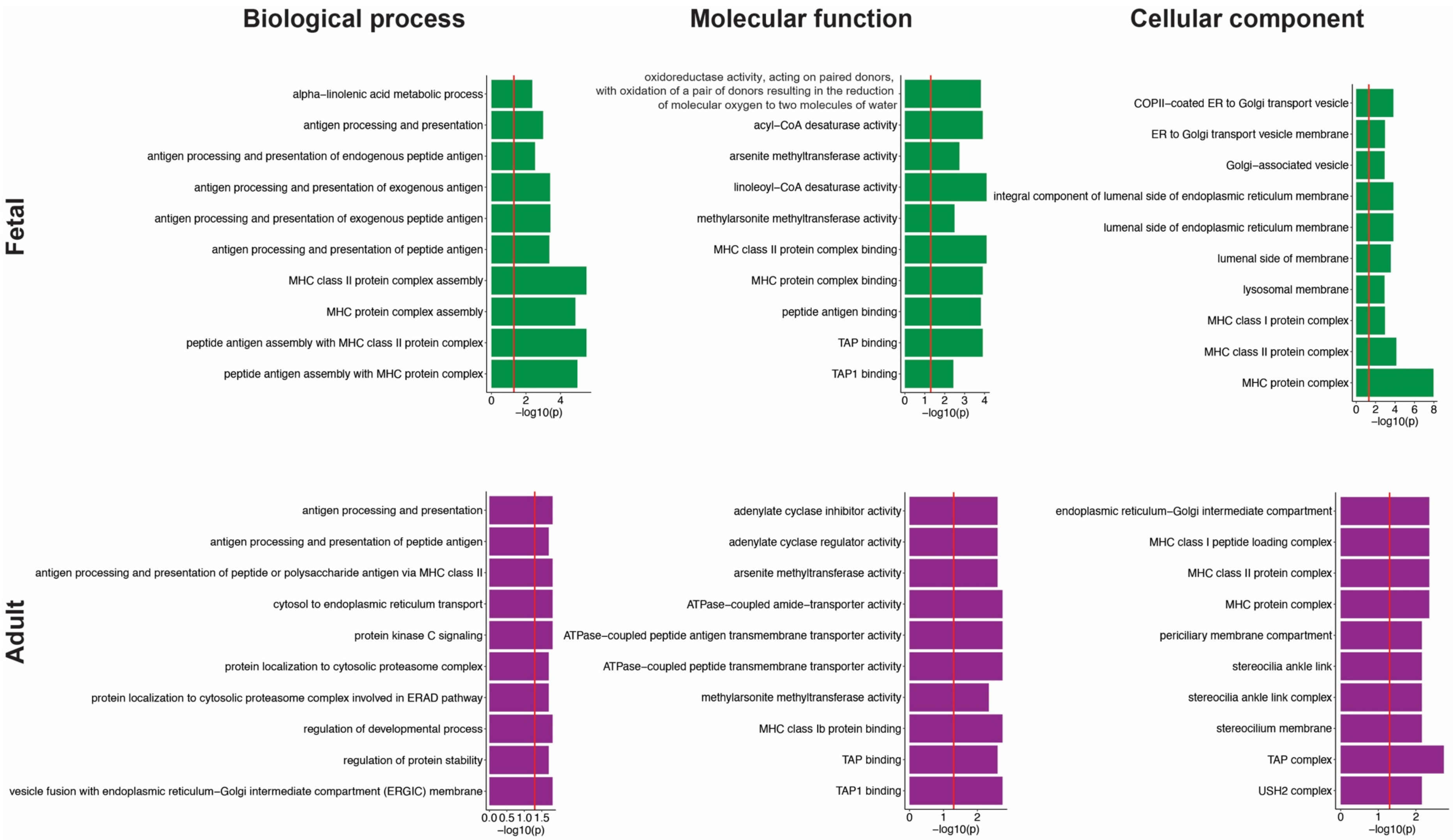

Supplementary Figure 4: Top 10 biological processes, molecular functions and cellular components for fetal and adult cortex-specific genes associated with the ASD-associated eQTLs. Gene ontology (GO) enrichment analysis was performed using g:Profiler. The threshold for significance (red line) is $p < 0.05$.
